# Case Report: Coronavirus Disease and Pulmonary Tuberculosis in Patients with Human Immunodeficiency Virus: Report of Two Cases

**DOI:** 10.4269/ajtmh.20-0737

**Published:** 2020-08-18

**Authors:** Luís Arthur Brasil Gadelha Farias, Ana Livia Gomes Moreira, Eduardo Austregésilo Corrêa, Cicero Allan Landim de Oliveira Lima, Isadora Maria Praciano Lopes, Pablo Eliack Linhares de Holanda, Fernanda Remígio Nunes, Roberto da Justa Pires Neto

**Affiliations:** 1 Escola de Saúde Pública do Ceará (ESP/CE), Fortaleza, Brazil;; 2 Hospital São José de Doenças Infecciosas (HSJ), Fortaleza, Brazil;; 3 Departamento de Saúde Comunitária, Faculdade de Medicina, Universidade Federal do Ceará (UFC), Fortaleza, Brazil

## Abstract

Coinfection of SARS-CoV-2/*Mycobacterium tuberculosis* (MTB) in patients with HIV/AIDS has not been previously reported. Here, we present two cases of coinfection of SARS-CoV-2 and MTB in patients with HIV. The first case is a 39-year-old patient who was admitted with a 7-day history of fever, myalgia, headache, and cough. The second patient is a 43-year-old man who had a 1-month history of cough with hemoptoic sputum, evolving to mild respiratory distress in the last 7 days. Both patients already had pulmonary tuberculosis and subsequently developed SARS-CoV-2 infection during the 2020 pandemic. Nonadherence to antiretroviral treatment may have been a factor in the clinical worsening of the patients.

## INTRODUCTION

COVID-19, caused by SARS-CoV-2, represents a new fatal disease mainly characterized by a respiratory illness.^1^ On March 11, 2020, the WHO officially declared the infection to have risen to a pandemic state.^2,3^ In Brazil, the first case of COVID-19 was confirmed on February 26, 2020, and community transmission was recognized on March 20, 2020.^4,5^ On May 24, 2020, there were almost 350,000 confirmed cases and approximately 22,000 deaths, with a mortality rate of 6.9%. The most affected states were São Paulo (82,161), Rio de Janeiro (37,912), and Ceará (35,595).^6,7^

The coinfection of SARS-CoV-2 and *Mycobacterium tuberculosis* (MTB) in patients with HIV infection is a matter of concern and has not been well studied. Here, we present two cases of triple coinfections (HIV/SARS-CoV-2/MTB) in patients admitted to Sao José Hospital of Infectious Diseases, Fortaleza, Ceará, Brazil. Both patients provided informed consent to publish their clinical data. Nasopharyngeal swab samples were used for COVID-19 diagnoses based on the amplification of the betacoronavirus *E* gene and the specific SARS-CoV-2 *RdRp* gene using PCR. Sputum samples were used for MTB diagnosis.

## CASE REPORT

### Case 1.

**Figure 1.**
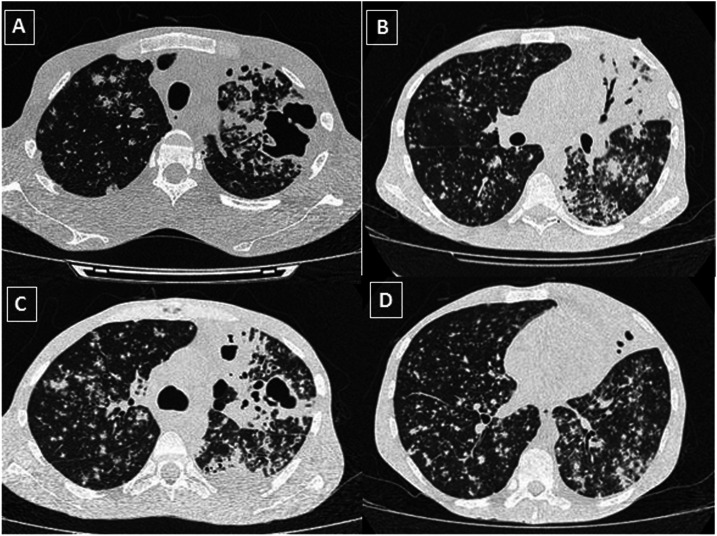
Chest computed tomography (CT). (**A**–**C**) Axial thin-section unenhanced CT image revealing a large cavitation with air bronchograms and consolidation. (**B**–**D**) Axial thin-section unenhanced CT image showing randomly distributed (miliary) nodules in both lungs associated with glass-ground opacities and consolidation in the left lower lobe.

A 39-year-old Italian man who had been living in Brazil for more than 20 years arrived at the emergency department (ED) in April 2020 with a known diagnosis of HIV/AIDS (HIV viral load 293,313 copies/mm^3^ and CD4 cell count 145/mm^3^) and a 7-day history of sudden fever (temperature 38.0°C), myalgia, headache, and cough. He had poor adherence to antiretroviral therapy and previous use of marijuana and crack. He denied recent travel. He also tested positive for the surface antigen of the hepatitis B virus. He reported previous tuberculosis (TB) treatment for 3 months, although he did not complete the scheme. On physical examination, he was cachectic (weight 38 kg), with mild respiratory distress, a heart rate of 108 bpm, a respiratory rate of 18 rpm, and blood oxygen saturation (SPO_2_) levels of 93% without supplementary oxygen. No lymphadenopathy was observed. A chest examination revealed bilateral crepitations, rhonchi, and wheezes. Chest computed tomography (CT) revealed a large cavitation located in the left lung associated with bilateral glass-ground opacities (Figure 1A–D). Blood cell count presented low hemoglobin (5.4 g/dL) and hematocrit levels (16.5%), lymphopenia (408/mm^3^), and normal platelet and white cell count ranges. The C-reactive protein level was markedly elevated (118.8 mg/L). Hepatic and renal functions were normal. D-dimer, troponin, aspartate transaminase (AST), alanine transminase (ALT), and ferritin levels were at normal range. Acid-fast bacilli smears were positive, and MTB DNA was detected using PCR (GeneXpert MTB/rifampicin [RIF] assay) without RIF resistance. During hospitalization, the patient experienced respiratory distress and required continuous oxygen via nasal cannula 2 L/minute during the first 3 days. Admission to the intensive care unit and invasive mechanical ventilation was not required. Treatment with isoniazid, ethambutol, pyrazinamide, and RIF was initiated. He was also treated with azithromycin (500 mg/day), hydroxychloroquine (HCQ) (400 mg/day) for 5 days, and ceftriaxone (2 g/day). Antiretroviral therapy was not started to avoid complications with anti-TB treatment. After being hospitalized for 3 weeks, the patient was clinically stable and discharged to home with outpatient follow-up. Antiretroviral therapy was planned to be initiated after the first 8 weeks of anti-TB treatment.^8^

### Case 2.

A 43-year-old Brazilian man arrived at the ED in May 2020 with a history of cough with hemoptoic sputum for more than 1 month, evolving to mild respiratory distress in the last 7 days. He did not show signs of a fever and other symptoms. He had a previous history of HIV/AIDS with no adherence to antiretroviral therapy, illicit drug abuse, and had generalized anxiety disorder. He had lost clinical and laboratory follow-up in the last 5 years. In 2015, the HIV viral load was 9,054 copies/mm^3^, and the T-CD4 cell count was 407/mm^3^. On physical examination, his weight was 55 kg, heart rate was 79 bpm, respiratory rate was 18 rpm, and SPO_2_ was 96%. No lymphadenopathy was observed. Blood cell count presented low hemoglobin (9.9 g/dL) and hematocrit levels (29.7%), and normal platelet and white cell count ranges. Lactate dehydrogenase and C-reactive protein levels were 312 U/L, above reference limits (140–271 U/L), and 52.3 mg/dL, respectively. The D-dimer level (0.6 mcg/mL) exceeded the normal range. Troponin, AST, ALT, and ferritin levels were in normal range.

**Figure 2.**
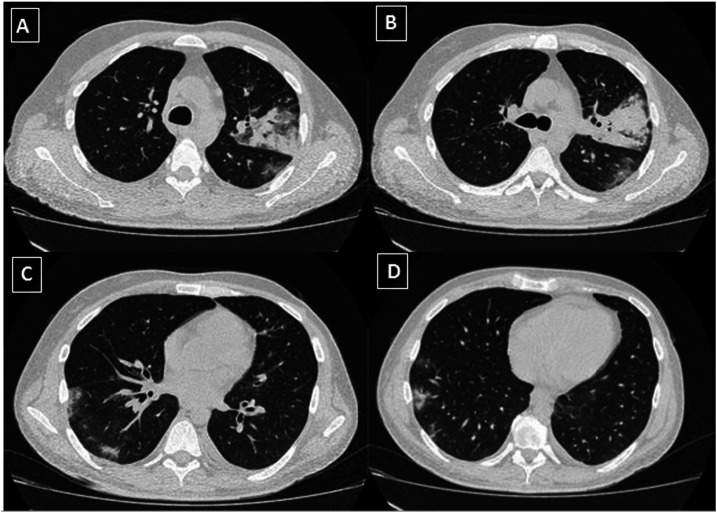
Chest computed tomography (CT). (**A** and **B**) Axial thin-section unenhanced CT image revealing consolidative pulmonary opacities in the left upper lobe. (**C** and **D**) Axial thin-section unenhanced CT image showing multifocal, rounded, and bilateral ground-glass opacities, with peripheral and posterior predominance in the right lower lobe.

Chest CT revealed bilateral glass-ground opacities occupying approximately 25% of both lungs (Figure 2A–D). Respiratory secretion was collected with a nasopharyngeal swab and tested positive for SARS-CoV-2. *Mycobacterium tuberculosis* DNA was detected using PCR (GeneXpert MTB/RIF assay) without RIF resistance. Treatment was initiated with azithromycin (500 mg/day), HCQ (400 mg/day) for 5 days, and ceftriaxone (2 g/day). He was treated with isoniazid, ethambutol, pyrazinamide, and RIF. Antiretroviral therapy was not started to avoid complications with anti-TB treatment. The patient was clinically stable and discharged to home after 1 week. Currently, the patient is under follow-up and remains asymptomatic without developing relapses.

## DISCUSSION

To the best of our knowledge, this is the first reported case of coinfection of SARS-CoV-2/MTB in patients with HIV/AIDS. HIV/MTB coinfection is prevalent worldwide—especially in low-income and developing countries such as Brazil—and its clinical consequences are well known. HIV patients with MTB latent infection have a higher risk of reactivation. Tuberculosis is the most common opportunistic disease and tends to be more lethal in cases of HIV. Extrapulmonary forms and disseminated disease occur more frequently in HIV, especially in those with high viral load and low T-CD4 cell count. However, as the COVID-19 pandemic develops, there is a concern regarding its clinical and epidemiological relevance in HIV/MTB–coinfected patients.

COVID-19 is an acute viral infection with frequent and severe pulmonary involvement, which can lead to hospitalizations and deaths. The lungs can be directly affected by the virus, leading to viral pneumonia. The immune system is also markedly affected and challenged by SARS-CoV-2. Leukopenia, lymphopenia, and an inflammatory cytokine storm are some of these immunological changes. Treatment with immunosuppressive drugs, such as corticosteroids, has been adopted in some cases. In this context, the impact of COVID-19 on HIV viral replication in HIV patients with MTB latent infection and in patients with HIV and active TB must be subjects of research. This includes the spectrum of clinical manifestations of the triple coinfections.

There are some reports that show that SARS/MTB and Middle East respiratory syndrome–CoV/MTB coinfections can augment other infections.^9^ One observational study on SARS-CoV-2/MTB coinfection concluded that MTB infection possibly increases COVID-19 susceptibility and severity.^10^ Blanco et al.^11^ recently published a case series of five HIV-infected patients with COVID-19, of whom none of them died, although two needed intensive care supportive therapy. Gervasoni et al.^12^ described 47 HIV-positive patients hospitalized by COVID-19 and concluded that HIV patients were not at greater risk of severe disease or death than HIV-negative patients. Motta et al.^13^ compared 69 patients with SARS-CoV-2/MTB coinfections, although they could not show TB as a major predictor of mortality in these patients. This appeared to be true in patients reported in the present study because even with HIV infection association, the patients did not evolve to a greater gravity. However, according to He et al.,^14^ patients with previous lung disease such as treated or untreated TB could affect the prognosis of patients with COVID-19, making greater vigilance necessary during outpatient follow-up.

The present article reports two cases of COVID-19 in patients with HIV/MTB coinfections. Both patients already had active TB before being infected with SARS-CoV-2. They both had a history of irregular antiretroviral treatment, detectable HIV viral loads, and low T-CD4 lymphocyte counts (< 500). Molecular tests (PCR and reverse transcription PCR) were positive for MTB and SARS-CoV-2 simultaneously. There was an overlap of symptoms commonly seen in active TB and COVID-19, such as cough and fever. One of the patients had hemoptoic sputum. Chest CT findings showed patterns that could be related to both diseases, although they cannot be differentiated from other pulmonary diseases such as bacterial pneumonia and chronic obstructive pulmonary disease.

Although the risk factors for COVID-19 still need to be fully understood, the two cases presented here may indicate that HIV/MTB coinfection could be another risk factor to be considered when evaluating SARS-CoV-2–infected patients. Nonadherence to antiretroviral treatment may have been a factor in the worsening of MTB/SARS-CoV-2 coinfection.^15^ The possibility of associated bacterial pneumonia could not be ruled out in both cases, and antibiotic coverage with ceftriaxone and azithromycin was chosen. Both patients were treated with HCQ. Thus, the usefulness of this drug is controversial. On June 17, 2020, based on evidence from the solidarity trial, U.K. recovery trial and a Cochrane review, WHO announced that the HCQ arm of the solidarity trial to find an effective COVID-19 treatment was being stopped. Both showed that HCQ does not result in the reduction of mortality of hospitalized COVID-19 patients.^16^ This study has some limitations. Herein, we studied only two cases of SARS-CoV-2 and MTB coinfection in HIV-infected patients. Interleukin-6 was not available at our center. It is important to carefully evaluate suspected SARS-CoV-2 patients in the presence of other infectious diseases, such as TB, especially if cohorting is performed for suspected SARS-CoV-2 to avoid nosocomial transmission.
